# Shots in Practice: An Audit of Depot Antipsychotics in Pakistan’s Outpatient Psychiatry Department

**DOI:** 10.1192/bjo.2025.10683

**Published:** 2025-06-20

**Authors:** Asma Suleman, Farakh Abdullah, Ali Anjum, Aneel Shafi

**Affiliations:** 1 Punjab Institute of Mental Health, Lahore, Pakistan; 2 Muhammad Nawaz Sharif Teaching Hospital, Lahore, Pakistan

## Abstract

**Aims:** The primary aim of this audit was to evaluate the adherence to the Maudsley Prescribing Guidelines for Long-Acting Injectable (LAI) antipsychotics in the outpatient department (OPD) setting at the Punjab Institute of Mental Health, Lahore, Pakistan. Specifically, the objectives were to:

Assess prescribing practices, including the use of test doses, baseline investigations, dose adjustments, dosing intervals, and combining LAIs with oral antipsychotics.

Identify gaps in clinical practices, such as patient monitoring and safety protocols, and their alignment with guidelines.

Highlight deviations like the use of LAIs in rapid tranquillization and high rates of polypharmacy.

Inform targeted interventions to improve patient safety and promote evidence-based care.

**Methods:** In July 2024, a random selection of 42 OPD prescriptions for patients receiving LAIs was reviewed. A structured proforma was designed to capture:

Patient demographics (age, gender).

Diagnosis,

Prescribing patterns (test doses, polypharmacy, and rapid tranquillization).

Safety protocols (baseline investigations, post-injection monitoring).

Patient education on medication side effects.

The proforma data facilitated a systematic evaluation of adherence to the Maudsley Prescribing Guidelines (14th edition) and provided insights into clinical practices at the Punjab Institute of Mental Health, Lahore.

**Results:** Demographics: The majority of the patients were male (76.2%) with a mean age of 41 years.

The majority of patients taking depot antipsychotics were diagnosed with schizophrenia (75.0%), followed by bipolar affective disorder (BAD) (21%), with a smaller proportion diagnosed with schizoaffective disorder (4%).

Prescribing Practices and Safety Protocols:

LAI usage: All patients received fluphenazine decanoate (25 mg intramuscular, biweekly), a first-generation antipsychotic (FGA).

Polypharmacy: All patients were concurrently prescribed a second antipsychotic, most commonly risperidone (4 mg/day).

Patient education: 78% of patients reported being informed about the side effects of injections. However, none was placed under post-injection observation, a significant gap in safety monitoring.

Test doses: No test doses were administered to patients receiving LAIs for the first time.

Rapid tranquillization: Fluphenazine injections were used in rapid tranquillization protocols, along with haloperidol (Serenace) and promethazine (Phenergan). This practice deviates from recommended guidelines.

Baseline investigations: No baseline ECG or other physical health assessments were conducted prior to initiating LAI therapy.

**Conclusion:** This audit highlights several critical gaps in the prescribing and monitoring practices for LAI antipsychotics at the outpatient department of the Punjab Institute of Mental Health. Key findings include the lack of test doses, absence of baseline physical health assessments (e.g. ECG), and insufficient post-injection observation. The use of fluphenazine in rapid tranquillization and the high prevalence of polypharmacy raise additional safety concerns.

Recommendations:

Staff education: Conduct regular training sessions on the Maudsley Guidelines, emphasizing test dose administration, baseline monitoring, and post-injection observation.

Test dose feasibility: Establish protocols to ensure test doses are administered before initiating LAI therapy.

Baseline investigations: Make baseline physical health assessments, including ECGs, mandatory for all LAI prescriptions to mitigate cardiac risks.

Review of tranquillization protocols: Revise rapid tranquillization protocols to align with evidence-based guidelines.

Polypharmacy monitoring: Regularly evaluate the rationale for combining multiple antipsychotics to minimize unnecessary polypharmacy.

Continuous audits: Perform regular audits to track improvements and identify persistent gaps in adherence to guidelines.

By implementing these measures, clinical practices can align more closely with international standards, ensuring safer and more effective care for patients receiving LAI antipsychotics.

